# Gel mobility shift scanning of pectin-inducible promoter from *Penicillium griseoroseum* reveals the involvement of a CCAAT element in the expression of a polygalacturonase gene

**DOI:** 10.1590/S1415-47572009005000021

**Published:** 2009-01-30

**Authors:** Andréa de O. B. Ribon, João Batista Ribeiro, Daniel B. Gonçalves, Marisa V. de Queiroz, Elza F. de Araújo

**Affiliations:** 1 Departamento de Bioquímica e Biologia Molecular, Universidade Federal de Viçosa, Viçosa, MGBrazil; 2 EMBRAPA Suínos e Aves, Concórdia, SCBrazil; 3 Departamento de Microbiologia, Universidade Federal de Viçosa, Viçosa, MGBrazil

**Keywords:** * Penicillium griseoroseum*, polygalacturonase, 5' upstream regulatory sequence, electrophoretic mobility shift assay

## Abstract

Previous reports have described *pgg2*, a polygalacturonase-encoding gene of *Penicillium griseoroseum*, as an attractive model for transcriptional regulation studies, due to its high expression throughout several *in vitro* growth conditions, even in the presence of non-inducing sugars such as sucrose. A search for regulatory motifs in the 5' upstream regulatory sequence of *pgg2* identified a putative CCAAT box that could justify this expression profile. This element, located 270 bp upstream of the translational start codon, was tested as binding target for regulatory proteins. Analysis of a 170 bp promoter fragment by electrophoretic mobility shift assay (EMSA) with nuclear extracts prepared from mycelia grown in pectin-containing culture medium revealed a high mobility complex that was subsequently confirmed by analyzing it with a double-stranded oligonucleotide spanning the CCAAT motif. A substitution in the core sequence for GTAGG partially abolished the formation of specific complexes, showing the involvement of the CCAAT box in the regulation of the polygalacturonase gene studied.

Polygalacturonases (PGs) are enzymes directed toward the degradation of D-galacturonic acid moieties of pectic substances. Several PG genes have been isolated from filamentous fungi, due to their importance in host-pathogen cross-talks and their industrial relevance, for example, in food and textile processing (Ribon *et al.*, 1999; Wubben *et al.*, 1999; Lang and Dörnenburg 2000; Jayani *et al.*, 2005). These genes are encoded by a multigene family and exhibit a high degree of polymorphism, resulting in enzymes with different biochemical properties, probably a reflection of fungus lifestyle in nature. The PG enzyme is characterized by the presence of eight amino acid residues that are strictly conserved among all groups of organisms and have been implicated in substrate binding and/or catalysis (van Santen *et al.*, 1999).

Fungal PGs are differentially regulated in response to carbon sources. Pectic components are the main inducers of PG genes, but expression is also observed in the presence of simple sugars and other sources, such as corn (Panda *et al.*, 2004). Glucose repression is observed in the majority of the genes studied so far, but there are reports of PG genes expressed constitutively regardless of the carbon source (Wubben *et al.*, 2000; Cotton *et al.*, 2003). In the phytopathogenic fungus *Botrytis cinerea*, the six PG genes characterized so far show different expression patterns, depending on the time and infected tissue (ten Have *et al.*, 2001). In addition, some recent reports confirmed that pH also triggers PG expression (Wubben *et al.*, 2000; Cotton *et al.*, 2003).

Despite the suggestion that pectic genes are subject to a general pectinolytic regulatory system, studies on the mechanisms involved in the transcriptional regulation of PG genes are scarce (de Vries *et al.*, 2002). Putative cis-elements have been described for all isolated PG genes, but there are only few data confirming their participation in gene expression. Among the sequences described, there are indications that CCAAT, SYGGRG or CCCTGA might play an important role in the transcription of PG genes, but no trans-acting factors that regulate genes coding for enzymes of the pectic system have been identified to date (Benen *et al.*, 1996; Ishida *et al.*, 1997; Parenicová *et al.*, 1998).

Previous reports have described *pgg2* (GenBank AF195113), a polygalacturonase-encoding gene of *Penicillium griseoroseum*, as an attractive model for transcriptional regulation studies, due to its high expression throughout several different growth conditions, even in the presence of non-inducing sugars such as sucrose, as opposed to *pgg1*, expressed only in pectin-containing medium, after 76 h of growth (Ribon *et al.*, 2002). *pgg2* is subject to catabolite repression by glucose. Nevertheless, *pgg2* transcripts are observed during mycelium cultivation in medium containing glucose and yeast extract, showing that these substances relieve the repression somehow. Since CCAAT has always been referred to as a binding motif for proteins that modulate expression of eukaryotic genes, it was assumed that this element could be important for the constant high-level gene expression observed in the previous work (Ribon *et al.*, 2002). Visual inspection of the 5' upstream gene region for cis motifs likely to represent binding sites for proteins that could explain the expression pattern seen revealed a CCAAT motif located at -270 bp to the translation start codon whose relevance for *pgg2* expression was studied in this work.

Nuclear extracts were prepared from *P. griseoroseum* (CCT 6421) mycelia grown for 24 h on minimal medium containing pectin as sole carbon source (Nagata *et al.*, 1993; Ribon *et al.*, 2002) and submitted to electrophoretic mobility shift assays (EMSA). As probe, a 170 bp fragment containing the putative CCAAT box was used. It was originated by enzyme cleavage of a 335 bp fragment amplified from plasmid pPG4.3 (*pgg2* gene clone) with forward primer 5' TGAGGAATGAATGAATGAATG 3' and reverse primer 5' GGCCATTCTAGACTAGGTGG 3'. The restriction generated products of 85 bp, 80 bp and 170 bp. The 170 bp fragment was purified from the agarose gel, using the Wizard SV Gel and PCR Clean-Up System (Promega, USA), and radiolabeled with [γ-^32^P]dATP (Sambrook and Russell, 2001). Labeled probes (5 ng) were incubated with nuclear extract at room temperature for 10 min in a total reaction volume of 20 μL containing 4 μL of 5X ligation buffer (200 mM KCl, 5 mM EDTA, 125 mM HEPES-KOH, pH 7.0, and 50% w/v glycerol). For non-specific competition assays, poly(dI-dC) was added to the reaction. Samples were analyzed by electrophoresis on a 4% non-denaturating polyacrylamide gel (acrylamide/bisacrylamide 19:1) at 100 V for 5 h, and then the gel was transferred onto Whatman 3 MM paper, covered with plastic film and exposed to BIOMAX MR film (Kodak) at -80 °C. Binding assays were also performed with synthetic oligonucleotides spanning the CCAAT motif (5'-TGATTT TCCAATGAGGGGTCC-3' and 5'-GGACCCCTCATTG GAAAATCA-3') and oligonucleotides altered at this site (5'-GATTTTCGTAGGAGGGGTCT-3' and 5'-AGACCC CTCCTACGAAAATC-3'). After annealing, the strands were labeled with [γ-^32^P]dATP using polynucleotide kinase (Promega). For competition assays, a 25- or 50-fold molar excess of the unlabeled oligonucleotide was added to the binding reaction.

When the 170 bp DNA fragment was used as probe, a band shift was observed, independently of the extract concentration employed in the binding reactions, which provides evidence that proteins in the extract recognized the CCAAT element, since it was the most probable cis-element present in the 170 bp fragment (Figure 1A). The experiment was also conducted with nuclear extracts prepared from mycelia grown in the same way described above, but originated from a different inoculum. Competition assays were performed with increasing concentrations of the non-labeled fragment, which explains the weaker band shifts observed (Figure 1B). However, an excess of the nonspecific competitor poly(dI-dC) did not eliminate band shift. When the electrophoretic mobility shift assay was repeated using the 23 bp double-stranded oligonucleotide as probe, gel retardation activity was again observed. Almost all specific protein-DNA complex formation was abolished upon the substitution of this fragment for a mutant-type oligonucleotide (Figure 1C).

Taken together, these results show that in the polygalacturonase gene studied the sequence CCAAT is responsible for the binding of protein complexes from induced *P. griseoroseum* mycelia that may be important for the expression *in vivo*. Since pectin and sucrose activate *pgg2* gene transcription in the initial 24 h of *in vitro* growth, it is reasonable to postulate that the CCAAT sequence may be involved in PG gene expression in other carbon sources as well. Sucrose is not a normal constituent of the pectin molecule, so gene activation may follow a different regulatory pathway not exclusive to the pectinolytic system, but possibly a general one controlled by a CCAAT element. We believe that this element regulates *pgg2* expression in pectin as well, but is not involved in the expression of the *pgg1* gene that is induced solely by pectin in 76 h of fungus growth (Ribon *et al.*, 2002). This hypothesis is consistent with the inexistence of a CCAAT sequence in the *pgg1* promoter (results not shown). CCAAT boxes have been implicated in the modulation of transcript levels in eukaryotes, and their functionality has also been reported for some genes that code for other cell wall degrading enzymes, such as cellulase and xylanase, and enzymes of biotechnological importance like amylase and penicillin (Raymondjean *et al.*, 1988; Litzka *et al.*, 1996; Zeilinger *et al.*, 1996; Zeilinger *et al.*, 1998; Tanaka *et al.*, 2000). Proteins binding to these sequences have been identified in *Aspergillus nidulans* and *Neurospora crassa* and resemble the Hap complex of *Saccharomyces cerevisiae* (Litzka *et al.*, 1996; Chen *et al.*, 1998; Steidl *et al.*, 1999; de Vries *et al.*, 2002). As far as we know, homologues have not yet been described in *Penicillium*.

Further studies are needed to determine the relationship between the binding of transcriptional regulators *in vivo* and gene expression level. Since the polygalacturonases are the most extensively studied enzymes in the pectinase family, we believe that new insights into the detailed mechanisms of expression of other polygalacturonase genes will be reported in the near future.

**Figure 1:**
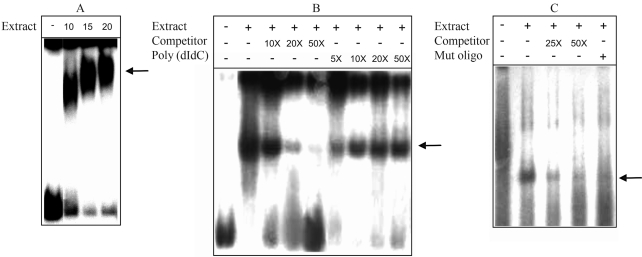
Electrophoretic mobility shift assays performed with nuclear extracts from *Penicillium griseoroseum* mycelia cultivated in media containing pectin as sole carbon source. The arrow indicates DNA-protein complexes. (A) A 170 bp DNA fragment containing the CCAAT motif (5 ng) was radiolabeled and used as probe in binding reactions with 10, 15 and 20 μg of nuclear proteins. (B) The same fragment was incubated with 10 μg of nuclear extract prepared from pectin-grown mycelia originated from a different inoculum. Specific competitor was added to the binding reaction at a 10-, 20- and 50-fold molar excess. Increasing molar excess of poly(dI-dC) was added as non-specific competitor. (C) Binding reaction mixture containing 10 μg of nuclear extracts and a 23 bp radiolabeled oligonucleotide harboring the CCAAT motif or a labeled oligonucleotide (Mut oligo) bearing point mutations in the CAAT element. In competition experiments, a 25- or 50-fold molar excess of unlabeled oligonucleotide was added to the binding reaction.
